# Supplementary material for: Induced abortion incidence and safety in Côte d’Ivoire
Source: PLoS One. 2020 May 7;15(5):e0232364. doi: 10.1371/journal.pone.0232364 (PMC7205243; doi:10.1371/journal.pone.0232364)
Supplement: Attachment — Submitted filename: Review R2.pdf [file pone.0232364.s004.pdf]

22 March 2020

Review of Manuscript: PONE-D-19-34624_R1

The revised manuscript has improved significantly; I really appreciate the authors for addressing most of the concerns that I raised in the first round of my review. However, there are serious problems in the calculation of abortion rates, based on the new description of calculation of abortion rates, which make the manuscript unpublishable if the authors cannot address them given the nature of data they have collected.

1) Lines 232-243. Please make it clear whether the number of abortions (each specific one: pregnancy removals, period regulations, et.) in the numerator of all of the rates you calculated is "life-time" incidences or only the abortions happened in the year preceding the interview. For example, when you say, "We calculated the one-year pregnancy removal incidence rate by determining the number of pregnancy removals reported in 2017 and in 2018 divided the number of pregnancy removals reported in 2017 and in 2018". Do you mean all pregnancy removals that a woman has had over her reproductive lifetime (say, from age 15 to the time of the interview) or the number of pregnancy removals that occurred over the year before the interview. If the number of incidences in the numerator of the calculated rate is "life-time" abortions, then your estimated rates cannot be one-year rate, you should use the number of incidences over the past year (before the date of the interview); in this case, you should have asked date of abortions in the questionnaire. Have you done this? If you clarify the structure of data that you have collected in the questionnaire can be helpful here; I don't see English translated copy of the questionnaire in the revised manuscript. How did you determined the number of abortions in 2017 or 2018?
   - From what you have written in Line *** ("The final incidence analyses involved bivariate and multivariable logistic regression to determine which characteristics were independently associated with experiencing an abortion in the year prior to the survey.", I gather that you have calculated the abortion rates for the abortions that happened in the 12-months before the interview. If this is the case, please revise your rates based on what I have discussed above.

2) Line 174-175. I see here you are saying, "We did not collect information about repeated abortions thus these details correspond to the most recent pregnancy removal or period regulation." So, did you include the most recent abortion incidence in the numerator of all incidence rates? If this is the case, therefore, you cannot claim that you have calculated "abortion rate" in this paper. Your study is limited to the pregnancy removal of the last pregnancy of women aged 15-49 in the study population. Following point 2 above, abortion rates refer to the total number of abortions that happen in a given period over the women-years of exposure to the risk of abortions in the same period. If you have not done this calculation, the section of abortion estimates is totally incorrect and unpublishable.

3) Lines 233-235. Following my pervious concern, you said that "To convert the proportion into a one-year incidence rate, we divided the estimate by the total number of years covered from

January 1, 2017 through the date of the interview." I am wondering how you can do this when the rate you calculated is based on the abortions that happened up to 2017 or up 2018. You cannot extend it to after 2017 or 2018. To calculate an annual rate of abortion, it is correct you divide the total number of abortions that happened in a give period by 1,000 woman-years of exposure to the risk of abortion in the same given period. For example, you should divide the total number of abortions that happened in the three-year period before the interview by the women-years of exposure to the risk of abortion in the same three years.
