# Supplementary material for: Induced abortion incidence and safety in Côte d’Ivoire
Source: PLoS One. 2020 May 7;15(5):e0232364. doi: 10.1371/journal.pone.0232364 (PMC7205243; doi:10.1371/journal.pone.0232364)
Supplement: Attachment — Submitted filename: PONE-ResponseToReviewers-v2-2020-02-20-sob.docx [file pone.0232364.s003.docx]

**PONE-D-19-34624 Response to Reviewer Comments**

Dear PLOS ONE Editorial Review Committee and Reviewers,

We would like to thank you for reviewing our manuscript and for the opportunity to revise and resubmit our work for further consideration at your esteemed journal. We appreciate the thoughtful and detailed feedback that the reviewers provided, and we have done our best to incorporate the recommended changes and additions. We feel these edits have strengthened our manuscript and hope you will agree. We look forward to hearing back from you regarding your final decision.

Best,

The Authors

**Journal Requirements**

When submitting your revision, we need you to address these additional requirements.

1. Please ensure that your manuscript meets PLOS ONE's style requirements, including those for file naming. The PLOS ONE style templates can be found at
<http://www.journals.plos.org/plosone/s/file?id=wjVg/PLOSOne_formatting_sample_main_body.pdf> and <http://www.journals.plos.org/plosone/s/file?id=ba62/PLOSOne_formatting_sample_title_authors_affiliations.pdf>

*We updated the file naming to be consistent with journal requirements. We believe the rest of the file is formatted in accordance with PLOS ONE style requirements.*

2. Please include additional information regarding the survey or questionnaire used in the study and ensure that you have provided sufficient details that others could replicate the analyses. For instance, if you developed a questionnaire as part of this study and it is not under a copyright more restrictive than CC-BY, please include a copy, in both the original language and English, as Supporting Information. Moreover, please include more details on how the questionnaire was pre-tested, and whether it was validated; and on the training of interviewers.

*We have incorporated more details into the methods section, which we describe below in response to the specific reviewer comments. We also uploaded the questionnaires in English and French with the resubmission.*

]3. Please provide additional details regarding participant consent. In the ethics statement in the Methods and online submission information, please ensure that you have specified (1) whether consent was informed and (2) what type you obtained (for instance, written or verbal, and if verbal, how it was documented and witnessed). If your study included minors, state whether you obtained consent from parents or guardians.

*Interviewers obtained informed, verbal consent from all participants, including minors, who were treated as adults in accordance with local IRB approval. More details and the specific new text that we added to clarify this protocol are provided in response to the specific reviewer comments below.*

4.PLOS requires an ORCID iD for the corresponding author in Editorial Manager on papers submitted after December 6th, 2016. Please ensure that you have an ORCID iD and that it is validated in Editorial Manager. To do this, go to ‘Update my Information’ (in the upper left-hand corner of the main menu), and click on the Fetch/Validate link next to the ORCID field. This will take you to the ORCID site and allow you to create a new iD or authenticate a pre-existing iD in Editorial Manager. Please see the following video for instructions on linking an ORCID iD to your Editorial Manager account: <https://www.youtube.com/watch?v=_xcclfuvtxQ>

*The corresponding author has an ORCID iD (*https://orcid.org/0000-0002-7650-5940*) but when she tried to add it to her PLoS ONE account, it says it is already linked to an account in the PLoS ONE system. Someone at PLoS ONE will need to merge her accounts (*[*suzannebell@jhu.edu*](mailto:suzannebell@jhu.edu) *and* [*suzanneobell@gmail.com*](mailto:suzanneobell@gmail.com)*).*

**Reviewer 1 Comments**

Incidence of abortion and safety estimates for Cote d’Ivoire
It is extremely important to come up with estimates of abortion and abortion safety for Cote d’Ivoire. I have questions about the methodology that can likely be addressed by the authors.

Abstract
There is a missing link between abortion is common and its contributing to maternal mortality. I think you need to add that it is often unsafe. Because many countries have high abortion rates and low abortion related morbidity and mortality.

*The reviewer makes a good point. We have modified a sentence in the background section of the abstract to clarify:“In Côte d’Ivoire, abortion is legally restricted unless a pregnancy threatens a woman’s life. Yet the limited available evidence suggests abortion is common and that unsafe abortion is contributing to the country’s high maternal mortality.”*

Why are these the first national estimates when you cite the Vroh study. Regardless, claims to primacy should not be in the title.

*The Vroh et al. study did not provide an abortion incidence. They only provided a lifetime prevalence and restricted the denominator to a population of women who had a child, so we could not determine the population rate from published findings. Upon the reviewer’s feedback, we changed the title to “Abortion incidence and safety in Cote d’Ivoire”.*

Intro
What do you mean by “unmet need”? Some estimates don’t actually ask women whether they want to be using a contraceptive. If this is the sort of measure you are using, find another. The high rate of unintended pregnancy may not be a result of unmet need for family planning but instead is a result of a low desire for childbearing.

*We were using the standard measure of unmet need from the DHS, which is an algorithm that estimates a population-level measure of unmet need using several related measures of risk of pregnancy, desire for a/another child, and use of contraception. While we acknowledge this measure is not without problems, it is a common measure in the field of demography and is useful in providing a population-level estimate that can be compared across time and geographies. However, given the reviewer’s concerns, we have removed reference to unmet need. With regard to the fact that more than one-third of pregnancies were unintended, we do not think this simply reflects a low desire for childbearing; fertility is high (total fertility rate is 5) and low desire for childbearing could be managed via contraception or avoiding sexual activity, so 35% of pregnancies being unintended is communicating something other than low desire for childbearing.*

Line 76. Is anyone ever punished? Reporting that providing abortions is punishable is not relevant if nobody is ever prosecuted for conducting an abortion. How can 47.9% of abortions take place in a health facility? Clearly what is on the law books is not the whole story.

*It is not uncommon for abortion to be illegal and punishable with jail time but for abortion to nonetheless be quite common. We do think it is important to describe the official law, regardless of enforcement, but we have added reference to the fact the punishment is rarely, if ever, enforced: “Despite a lack of evidence of enforcement, anyone who provides or assists in providing an abortion—as well as the woman who obtains an abortion herself—can be punished under the law with a prison sentence and fine.”*

Define unsafe on line 80.

*We added the WHO definition to the second half of that sentence to clarify: “West Africa has some of the highest rates of unsafe abortion in the world, with estimates indicating as many as 85% of abortions in the region are unsafe, which the World Health Organization defines as being performed by an individual lacking the necessary training or in an environment not conforming to minimal medical standards.”*

Methods
The description of PMA 2020 as doing research on smartphones is very confusing. Did they do that for this study? Later it says face-to-face interviews.

*PMA interviewers use smartphones to implement the survey, but the respondent is not self-administering the questionnaire. We have added the second half of this sentence to clarify: “All women age 15 to 49 were eligible to participate in the face-to-face surveys, which interviewers conducted in French or local languages using smartphones, entering data via an Open Data Kit (ODK) application on the phone.”*

How does the identification of a confidante work? I would like to know what question is asked – do you have a friend with whom you talk about personal matters like sex and pregnancy?

*We describe the confidante identification process and definition in the second paragraph of the Measures section within the Methods section, but we have made it more explicit that the interviewer defined the confidante relationship to the respondent: “In the abortion module, the interviewer first asked the respondent to indicate the number of close female confidantes she had, defining a close female confidante as a woman age 15 to 49 currently living in Côte d’Ivoire who shares personal information with the respondent and with whom the respondent shares personal information.”*

It seems very difficult to get a woman to describe the procedure that was done to her in an abortion well enough to know if the technique was sound. And certainly impossible that she should know this about a confidante’s abortion. Reports of higher unsafety among confidantes seems likely due to women just not knowing. Women don’t even necessarily know what pills they are given. How were these questions asked? “Surgery” is definitely a confusing term. I hope that wasn’t used. Real medication abortion pills (miso and mife) are safe even when provided by someone untrained.

*The reviewer makes a good point regarding not knowing the specific type and clinical context of the reported surgery, which we identify as a limitation: “Women were unable to provide details on the specific surgery or training of their provider, nor were many able to provide details about the specific pills they took. This could result in misclassification in both directions (e.g. facility-based surgery that was actually performed by an untrained provider, medication abortion drugs categorized as “other pills”), which would reduce the likelihood of systematic error in the end results.”*

*Regarding the “surgery” term, interviewers were not reading that option (or others) aloud; they were only selecting it if the woman described having a procedure from a provider. In the Measures section of the Methods section, we added the following additional detail: “Interviewers did not read method and source options aloud; women volunteered their answer and interviewers probed when necessary to determine the appropriate response option to select. In the pilot, women were unable to provide detail regarding the specific surgery type (e.g. dilation and curettage, manual vacuum aspiration), thus interviewers only selected the one “surgery” option if a woman described having a medical procedure.”*

*We agree with the reviewer that the higher level of unsafe abortions among confidantes may be related to lack of knowledge about the more safe abortions (that don’t result in complications). We explain as much in the Discussion: “Using our incidence estimate and data on the Côte d’Ivoire population, we estimate there were more than 225,000 induced abortions in 2017, the majority of which were unsafe (62.4% for respondents and 78.4% for confidantes). Since more complicated abortions are likely to be more visible to one’s social network, we view the confidante safety estimate as an overestimate. Thus, the respondent finding that 62.4% of abortions are most unsafe is more accurate.”*

Why was the term “pregnancy removal” used and what does it mean? Does it include miscarriage management?

*During the pilot, we tried to determine more descriptive terminology to refer to abortion instead of simply using the direct translation of “induced abortion”, which is a more stigmatizing or triggering term/phrase. We did not intend for it to include miscarriage management, although it might have. To reduce the likelihood of this, we had an initial preamble in conjunction with the first question of the abortion section that made clear we were talking about “pregnancy removal” in the context of an unintended pregnancy (“Sometimes women are worried they are pregnant or get pregnant when they do not want to be and they do something to remove the pregnancy.”). We added more detail about the piloting and intent of this phrase in the Methods section: “Terminology and question phrasing are particularly important when addressing a sensitive topic like induced abortion. In order to avoid inclusion of miscarriage experiences, interviewers read the following preamble at the outset of the abortion module to indicate that subsequent questions were in the context of an unintended pregnancy: “Sometimes women are worried they are pregnant or get pregnant when they do not want to be and they do something to remove the pregnancy.” To assess respondent interpretation, the pilot questionnaires included quantitative face validity questions where the interviewer asked the respondent to describe how she interpreted or understood the phrases “pregnancy removal” and “period regulation at a time when you were worried you were pregnant”. In total, interviewers conducted 31 pilot surveys in Cote d’Ivoire. Interviewers indicated that 100% of pilot survey respondents the “pregnancy removal” and “period regulation” phrases correctly.”*

*We also added reference to the possibility that we capture some miscarriages in the limitations of the Discussion section: “Lastly, although pilot results indicated correct interpretation of the pregnancy removal and period regulation phrases, the novel framing of these questions could be unintentionally capturing some miscarriages.”*

Some women (apparently) don’t have confidantes. If this is real and not just an unwillingness to disclose to your interviewer, then these people are not represented in your confidante-based estimates. Do they have higher abortion rates than women with confidantes? If so, your estimate of confidante abortions is underestimated because the type of woman who has no confidantes is not represented. Or vice versa if their abortion rate is lower. Why not have the confidante’s data included along with those with no confidante. Otherwise the sort of people who don’t have friends aren’t represented. How do those with no confidantes differ in terms of willingness to disclose other sensitive info, socioeconomic circumstances, unintended pregnancy risk? It seems important to know who is omitted in the confidante estimates.

*The reviewer brings up an important potential bias, which we tried to address in the analysis (using a similar approach to what the reviewer suggested, in fact!). We have a separate confidante methods paper that provides a far more detailed description of the confidante method assumptions and adjustments taken to account for violations of method assumptions, which is currently under review. However, an early version of the paper was submitted at a conference last year and is available online (See Bell, SO. 2019. Methodological Advances in Survey-Based Abortion Measurement: Promising Findings From Nigeria, India, and Cote d’Ivoire. Population Association of America Annual Meeting, Austin, TX. Available at:* [*http://paa2019.populationassociation.org/uploads/191027*](http://paa2019.populationassociation.org/uploads/191027)*). We have provided more detail on these adjustments in the analysis section of the Methods section in the revised submission. In summary, we found that confidantes had somewhat different characteristics (likely as a result of the “missing” confidantes that correspond to the 35% of respondents who reported no confidante). We essentially imputed the likelihood of these missing confidantes having had a recent abortion using a Poisson model that regresses the characteristics of the respondents who had no confidantes, which is essentially what the reviewer is recommending; we seem to have had the same idea but hadn’t described it sufficiently! It is important to account for these “missing” confidantes as we found the respondents who had no confidantes had significantly different characteristics and were less likely to have reported their own abortion. We also constructed post-stratification weights using the respondent characteristics as a reference point in order to get the surrogate sample (i.e. the confidante data) to be representative of women of reproductive age.*

Why would the estimate of safety differ by whether it was a respondent or confidante based estimate? Is it because you learn more about the methods and training of the provider when it is the woman’s own abortion? Or is it because you only learn about your confidante’s abortion when it results in a complication?

*We have thought critically about the direction of the bias in comparing the respondent and confidante safety estimates. A priori, we thought the confidante estimates suffer from bias in the direction of overestimating unsafe abortions because respondents (and people generally) would be more likely to know about confidante’s abortions that resulted in complications and perhaps required the involvement and support of more people in the process of terminating the pregnancy and receiving treatment; our results support this hypothesis. Very few respondents reported a confidante’s abortion but were unable to report the method or source so that is unlikely to be the reason the confidante estimate of unsafe abortion is higher. The first paragraph of the discussion details this interpretation: “Using our incidence estimate and data on the Côte d’Ivoire population, we estimate there were more than 225,000 induced abortions in 2017, the majority of which are unsafe (62.4% for respondents and 78.4% for confidantes). Since more complicated abortions are likely to be more visible to one’s social network, we view the confidante safety estimate as an overestimate. Thus, the respondent finding that 62.4% of abortions were most unsafe is more accurate.”*

An annual rate of 39.9 abortions per 1000 women is not the same as 4% of women getting an abortion if the rate includes multiple abortions per woman. Does it?

*We did not ask about repeated abortions for the respondent or the confidantes. So the 4% is an accurate prevalence based on the available data. We added a sentence to the Methods section to clarify: “We did not collect information about repeated abortions thus these details correspond to the most recent pregnancy removal or period regulation.”*

Typo Page 17 35.9% reported no confidantes. Not no abortions. This seems very high, by the way. What question was asked where more than a third of women have no close friends.

*Thanks for catching the typo. We have addressed it. And yes, we were similarly surprised by this. We added additional clarity around the confidante definition (“a close female confidante as a woman age 15 to 49 currently living in Côte d’Ivoire who shares personal information with the respondent and with whom the respondent shares personal information”). We think the requirement around mutual sharing of personal information may have biased the relationship and caused women who do have close female friends to not report them thinking they do not meet this criterion. We raise the concern in our discussion of the limitations: “Another concern is that 35.9% of respondents reported no confidantes. We sought to counteract potential selection bias in the confidante data generated by the 64.1% who did report at least one confidante, however, biases may remain. Further work is needed to determine the best confidante relationship definition that optimizes representativeness of the surrogate confidante sample and respondent knowledge of confidante’s abortion experiences.” In future work, we want to experiment with using this phrase and a simple “closest friend” phrase to see how confidante results differ.*

I think it is problematic that you do not compensate women for their time to participate. If you do, change “volunteered” their time.

*Households received a small amount of phone credit (1000 CFA, which is approximately 1.6 USD), but individual women within the household did not receive an incentive for their participation. In the acknowledgement, we now say, “…as well as the women who took time to participate.”*

**Reviewer 2 Comments**
Review of Manuscript: PONE-D-19-34624
The manuscript entitled “The first national abortion incidence and safety estimates for Cote d'Ivoire” aims to estimate induced abortion directly and indirectly and examines correlates of unsafe abortion, based on a population-based survey of women of reproductive age. The manuscript is largely a descriptive study and has the potential to contribute to the literature. However, it suffers from the below important methodological and structural issues.

1) Lines 104-124. The method of data collection, the structure of abortion data have not been clearly and precisely described. The authors need to provide a detailed description of method of data collection, structure of the questionnaire, sampling, and representativeness of the sample, and the survey method. I suspect that method of “smart phones” has covered all the study population; because many people are likely that they do not have a cellphone. Therefore, most likely the sample is not representative.

*Sorry about the confusion; we obviously needed to clarify aspects of our sampling design! We used the same approach as the DHS, relying on the statistical bureau to select enumeration areas (clusters) using probability proportional to size sampling within sampling strata (i.e. urban/rural) and generating weights that represent the inverse of the probability of selection. Sampling had nothing to do with having a cell phone. Interviewers mapped and listed all households in selected clusters and invited women aged 15 to 49 in sampled households to participate. Interviewers used smartphones to conduct the face-to-face interview; respondents did not need a phone to participate, nor did they self-administer the questionnaire on a phone. This section of the methods description now reads as follows: “The sampling strategy relied on an urban-rural stratified cluster design with probability proportional to size selection of 73 enumeration areas (EAs), each of which represented a cluster of approximately 200 households. The National Statistics Institute (INS) selected the EAs from a sampling frame provided by the 2014 General Census of Population and Housing. In each EA, female resident interviewers mapped and listed all households and supervisors randomly selected 35 households from each EA sampling frame created. All women age 15 to 49 identified in selected households were eligible to participate in the face-to-face surveys, which interviewers conducted inn French or local languages using smartphones, entering data via an Open Data Kit (ODK) application on the phone;* *the English and the French translation of the questionnaire are provided in the supplementary materials (S1–S2). In order for the data to be nationally representative we constructed survey weights, which we calculated using the inverse of the probability of selection, accounting for the probability of EA selection, probability of household selection, and household and female response rates. The final sample included 2,738 de facto women (female response rate 98.1%). The Johns Hopkins Bloomberg School of Public Health and the Comité d'Éthique de la Recherche of Côte d’Ivoire provided ethical approval for this study. Women provided verbal informed consent prior to participation, with minors treated as adults in accordance with local IRB approval. Interviewers indicated receipt of verbal consent by checking a box in the smartphone survey to confirm receive of consent and entering their name as a witness to the consent process.” We hope this additional detail has addressed the reviewer’s concerns.*

2) Lines 127-169: The authors’ description of “measures” of abortion rates are not at all precise, clear and useful. It is not clear how the abortion incidence rates have been calculated and what kind of data structure have been used to estimate one-year incidence rate and so on. The authors need to write in details and with precision how one-year pregnancy removal rate, one-year abortion incidence rate, combined pregnancy removal/period regulation rate were calculated.

*We have restructured the analysis section and provided additional details on the abortion incidence calculation to improve clarity. Hopefully this addresses the reviewer’s concerns.*

*“We conducted univariate analyses of respondent and confidante characteristics. Due to questionnaire length constraints, we only collected information on confidantes’ age and education. We made a number of adjustments to the confidante data to improve the validity of the abortion incidence and safety estimates. The confidante estimates included all confidante pregnancy removals and period regulations that respondents reported with certainty (response option “Yes, I am certain”) or less certainty (response option “Yes, I think so”) when they could still provide the specific method(s) the confidante used. Inclusion of the less certain abortions helped to counteract respondent’s incomplete knowledge of their confidante’s abortions (i.e. transmission bias). We also adjusted the confidante estimates for potential selection bias resulting from the fact that some respondents reported zero confidantes. For this adjustment, we ran a Poisson model to predict the likelihood of these “missing” confidantes having had an abortion in the prior year. This model regressed the socioeconomic characteristics of the confidantes and the respondents with no confidantes on the available confidante abortion incidence data. We then used the model to predict the likelihood of the “missing” confidantes having had a recent abortion based on the corresponding respondents’ characteristics. We used this information to create a new variable that combined respondent reported confidante abortion data for those with confidantes, and the predicted probability of abortion in the prior year for the confidantes who were not in the sample because they had no close friends who we could have captured in the respondent sample. Research on mortality rate estimation using survey data has employed a similar modeling approach [16]. To ensure these confidante data had characteristics that reflected the population of reproductive aged women in Cote d’Ivoire, we constructed post-stratification weights using the weighted respondent data distributions as the reference.*

*We calculated the one-year pregnancy removal incidence rate by determining the number of pregnancy removals reported in 2017 and in 2018 divided by the number of women in each sample. To convert the proportion into a one-year incidence rate, we divided the estimate by the total number of years covered from January 1, 2017 through the date of the interview. We then multiplied the value by 1,000 to generate the one-year estimate of pregnancy removal per 1,000 women age 15 to 49. We scaled the standard errors in the same manner. We also calculated the combined pregnancy removal and period regulation one-year incidence rate (which we refer to as “likely-abortion”). To calculate the final one-year incidence rates of induced abortion, we averaged the pregnancy removal rate and the likely-abortion rate. We averaged the two point estimates because we believe the pregnancy removal data fails to capture some abortions (that women may not view as abortions or are not willing to admit are abortions) while the period regulation data likely includes some experiences that we would not consider to be abortions. We generated all these estimates separately for respondents and confidantes.*

*We calculated one-year pregnancy removal incidence rates, likely-abortion incidence rates, and the associated averages overall and by age and education for respondents and confidantes; we also calculated these rates by residence and wealth for respondents, for whom we had data on these characteristics. The final incidence analyses involved bivariate and multivariable logistic regression to determine which characteristics were independently associated with experiencing an abortion in the year prior to the survey. We used the unadjusted confidante likely-abortion dichotomous incidence data in order to conduct the logistic regression as the model assumes a Bernoulli distributed outcome variable whereas the Poisson predicted confidante incidence variable is continuous.”*

3) The manuscript includes several levels of analysis of abortion: abortion rate estimates and correlates, and multivariate analysis of unsafe abortion. Instead of having an undeveloped manuscript with different mixed results, I would suggest the authors to break down this manuscript into two separate manuscripts: 1) estimating abortion rates; 2) studying unsafe abortion.

*While we understand this is a lot of information, we feel it is important to include both incidence and safety estimates in this manuscript as they are related measures that together provide a comprehensive picture of abortion in Cote d’Ivoire. We hope we have laid out the content coherently, in a manner that allows the reader to easily follow the information. On a more practical level, this is a multi-country study and we do not have sufficient project funds to separate each of these country specific abortion papers into two while ensuring open access, which is a priority for us.*
