# Supplementary material for: Induced abortion incidence and safety in Côte d’Ivoire
Source: PLoS One. 2020 May 7;15(5):e0232364. doi: 10.1371/journal.pone.0232364 (PMC7205243; doi:10.1371/journal.pone.0232364)
Supplement: Attachment — Submitted filename: PONE-ResponseToReviewers2-v2-2020-04-07-sob.docx [file pone.0232364.s005.docx]

**PONE-D-19-34624 Response to Reviewer Comments: Round 2**

Dear PLOS ONE Editorial Review Committee and Reviewers,

We would like to thank you for a second opportunity to revise and resubmit our manuscript. We believe we have clarified and addressed the reviewer concerns and look forward to hearing back from you regarding the final decision.

Best,

The Authors

**Reviewer 2**

The revised manuscript has improved significantly; I really appreciate the authors for addressing most of the concerns that I raised in the first round of my review. However, there are serious problems in the calculation of abortion rates, based on the new description of calculation of abortion rates, which make the manuscript unpublishable if the authors cannot address them given the nature of data they have collected.

1) Lines 232-243. Please make it clear whether the number of abortions (each specific one: pregnancy removals, period regulations, et.) in the numerator of all of the rates you calculated is “life-time” incidences or only the abortions happened in the year preceding the interview. For example, when you say, “We calculated the one-year pregnancy removal incidence rate by determining the number of pregnancy removals reported in 2017 and in 2018 divided the number of pregnancy removals reported in 2017 and in 2018”. Do you mean all pregnancy removals that a woman has had over her reproductive lifetime (say, from age 15 to the time of the interview) or the number of pregnancy removals that occurred over the year before the interview. If the number of incidences in the numerator of the calculated rate is “life-time” abortions, then your estimated rates cannot be one-year rate, you should use the number of incidences over the past year (before the date of the interview); in this case, you should have asked date of abortions in the questionnaire. Have you done this? If you clarify the structure of data that you have collected in the questionnaire can be helpful here; I don’t see English translated copy of the questionnaire in the revised manuscript. How did you determined the number of abortions in 2017 or 2018?

- From what you have written in Line *** (“The final incidence analyses involved bivariate and multivariable logistic regression to determine which characteristics were independently associated with experiencing an abortion in the year prior to the survey.”, I gather that you have calculated the abortion rates for the abortions that happened in the 12-months before the interview. If this is the case, please revise your rates based on what I have discussed above.

*Our paper reports on the one-year abortion incidence rate, not lifetime incidence. The numerator for each rate is the number of events (i.e. pregnancy removal, period regulations, or the combination) in 2017 and 2018 up to the date of the interview. However, the denominator accounted for the additional 0.55 year in 2018 as we divided by the total number of woman-years so that the result, when multiplied by 1,000, represents the approximate one-year incidence rate per 1,000 women of reproductive age. We clarified this approach in the methods section: “We separately calculated the one-year pregnancy removal and period regulation incidence rates. Since we were unable to collect data on month of the event, we included events from 2017 through the date of interview in 2018. To convert this to an annualized one-year incidence rate, we divided the number of events in in 2017 and 2018 by the number of woman-years between January 1, 2017 through the date of the interview in 2018; each respondent contributed on average 1.55 woman-years. We then multiplied the value by 1,000 to generate the one-year incidence rate per 1,000 women age 15 to 49. We scaled the standard errors in the same manner.”*

*Since we cannot do a similar adjustment in the case of the bivariate and multivariate analyses, we reframed the bivariate/multivariate logistic regression analysis as follows to be more accurate: “The final incidence analyses involved bivariate and multivariable logistic regression to determine which characteristics were independently associated with experiencing a recent abortion in the approximately one year prior to the survey (2017 through beginning of 2018).”*

*The English version of the questionnaire should be attached as a supplemental file for review .*

2) Line 174-175. I see here you are saying, “We did not collect information about repeated abortions thus these details correspond to the most recent pregnancy removal or period regulation.” So, did you include the most recent abortion incidence in the numerator of all incidence rates? If this is the case, therefore, you cannot claim that you have calculated “abortion rate” in this paper. Your study is limited to the pregnancy removal of the last pregnancy of women aged 15-49 in the study population. Following point 2 above, abortion rates refer to the total number of abortions that happen in a given period over the women-years of exposure to the risk of abortions in the same period. If you have not done this calculation, the section of abortion estimates is totally incorrect and unpublishable.

*The reviewer brings up an important limitation that much of the survey-based abortion literature in low-resource settings also has. We know of no national estimates of repeat abortion and associated timing in Cote d’Ivoire. Data from the US suggests 48% of abortions are repeat, but the average duration between repeat abortions is 44 months (see Jones et al, 2006. Repeat abortion in the US: Occasional Report no. 29). Thus, while this would result in our incidence estimates being biased downward, we do not think the impact would be substantial. The methods section already clarifies that our data do not account for repeat abortion, but we added a sentence to the discussion section to explicitly describe this limitation: “... However, our rates do not include repeat abortions over the 2017/2018 period, thus to the extent that Ivoirian women are having repeat abortions in quick succession, our rates would be underestimates.”*

*To the reviewer’s other comment regarding abortion incidence calculation, see our prior response.*

3) Lines 233-235. Following my pervious concern, you said that “To convert the proportion into a one-year incidence rate, we divided the estimate by the total number of years covered from January 1, 2017 through the date of the interview.” I am wondering how you can do this when the rate you calculated is based on the abortions that happened up to 2017 or up 2018. You cannot extend it to after 2017 or 2018. To calculate an annual rate of abortion, it is correct you divide the total number of abortions that happened in a give period by 1,000 woman-years of exposure to the risk of abortion in the same given period. For example, you should divide the total number of abortions that happened in the three-year period before the interview by the women-years of exposure to the risk of abortion in the same three years.

*Our responses to the reviewer’s prior comments have sought to clarify how we calculated the annualized one-year incidence estimates, dividing by the total woman-years over the same period as the numerator, with each woman in the sample contributing on average 1.55 years. We hope this has addressed the reviewer’s methodological concerns.*
